# Prevalence of Cannabis Use Disorder and Associated Factors among Cannabis Young Adult Users at Shashemene Town, Oromia Region, Ethiopia, 2016

**DOI:** 10.1155/2018/6731341

**Published:** 2018-02-19

**Authors:** Mikiyas Tullu, Telake Azale, Dessie Abebaw, Haddis Solomon, Yodit Habtamu

**Affiliations:** ^1^Amanuel Mental Specialized Hospital, Addis Ababa, Ethiopia; ^2^Department of Public Health, College of Medicine and Health Sciences, University of Gondar, Gondar, Ethiopia; ^3^Department of Epidemiology, College of Health Science, Jimma University, Jimma, Ethiopia

## Abstract

**Introduction:**

Cannabis users are at high risk of developing cannabis use disorder which is a problematic pattern of cannabis use leading to clinically significant impairment or distress.

**Objective:**

To assess the magnitude of cannabis use disorder and associated factors among young adults using cannabis at Shashemene Town, Oromia Region, Ethiopia.

**Methods:**

A cross-sectional study was conducted at Shashemene Town, from May to June 2016. Young adults aged 18–25 who use cannabis and are permanent residents of Shashemene were included in the study. Using single proportion formula, 423 participants were selected using exponential discriminative snow ball sampling.

**Result:**

This study revealed that the magnitude of cannabis use disorder was 42.2%. The most contributing factors that remained to be statistically significant with cannabis use disorder were common mental disorder (AOR = 2.125, 95% CI: 1.218, 3.708), current cigarette smoking (AOR = 6.118, 95% CI: 2.13, 17.575), and current use of shisha (AOR = 4.313, 95% CI: 2.427, 7.664).

**Conclusion:**

The magnitude of cannabis use disorder among young adults using cannabis was high.

## 1. Background

Cannabis use disorder is a problematic pattern of cannabis use which leads to impaired control over cannabis use and difficulty in ceasing use despite its harm [1]. Drug abuse is a global phenomenon affecting almost every country, with cigarettes, cannabis, and alcohol being the most commonly used and abused substances. Among them cannabis is illegal. As compared to other psychoactive substances cannabis, most commonly known as marijuana, is the most widely used drug worldwide [2].

Even though there is no international consensus, young adulthood is a period where transition takes place from childhood to adulthood. Arnett et al. defines this group as “emerging adults” from 18 to 25 years, those who are neither children nor adults and who are in between with their own identity and behavior. It is the age of instability, self-focus, feeling in between, and possibilities [3–5]. Young adults are the most vulnerable group of people than any age group to cannabis dependence and related problems which produce more years lived with disabilities [5]. In 2013, cannabis was used by 80.6% of current illicit drug users in the United States [6].

Globally 2% cause-specific disability-adjusted life years (DALYs) for young people are attributed to illicit drug including cannabis [7]. The study examined morbidity, mortality, and economic costs attributable to the abuse of alcohol, illegal drugs, and tobacco in Canada for the year 2002 and revealed that cannabis and other illegal drugs accounted for approximately $8.2 billion of the nearly $40 billion cost of substance abuse in Canada in 2002 [8].

The probability of cannabis addiction in heavy or daily user is enormous. The vulnerability increases in adolescents whose risk of addiction is 16%, while adults have 5–10% risk of becoming addicted [9]. Canadian community health survey reported that the prevalence of cannabis dependence among adolescent and young adult in 2012 was 5% [10]. A three-year prospective study in the Netherlands on high-risk young adults reporting heavy use in 2013 found that nearly 40% developed cannabis dependence [11].

Another study in Holland revealed that cannabis dependence was 42% [12]. In 2013 the prevalence of cannabis abuse or dependence was 7.4% among youth in USA and the rate was about half (3.55) among adolescents [6]. National Survey on Drug Use and Health (NSDUH) revealed that cannabis was the illicit drug with the largest number of persons with past-year dependence or abuse in 2013. Of the 6.9 million persons aged 12 or older who were classified with illicit drug dependence or abuse in 2013, 4.2 million persons had cannabis dependence or abuse (representing 1.6% of the total population aged 12 or older and 61.4% of all those classified with illicit drug dependence or abuse [13]). Another study conducted in USA reported that 38.5% of daily cannabis users met criteria for cannabis dependence [14]. According to Fergusson and Boden 20% of young-age people will have used cannabis at least once and among the users 10% develop cannabis dependence [15].

A longitudinal cohort study conducted in Australia in 2002 among young adults shows 7% prevalence of cannabis dependence according to DSM-IV criteria for cannabis dependence [16]. A longitudinal study in India conducted by Kumar et al. in 2013 showed that cannabis is abused by 3.26% of the study population, that is, about in the range of cannabis use reported from different states of India which is from 4–20% [17].

A community household-based survey with cross-sectional design in Rwanda that aimed to determine the prevalence of cannabis dependence among adolescent and young adults shows 2.54% prevalence of cannabis dependence [18].

A recently published (2015) cohort study which considered cannabis abuse and dependence as cannabis use disorder (CUD) showed the lifetime prevalence of cannabis use disorder to be 19.1%, with an average age of onset of 18.6 years [19].

Key risk factors for later illicit drug use and cannabis disorder included male gender; family-related factors including parental use of illicit drugs and exposure to childhood sexual abuse; individual factors including novelty-seeking behavior, conduct disorder, and use of alcohol or tobacco; and affiliation with substance use peers [16, 20–23].

Cannabis availability, regular use of cannabis, peer pressure, and common mental disorder were factors having significant association with cannabis use disorder in different studies [22, 24–28].

Gateway hypothesis which was developed by Kandel explained that the sequence of drug use occuring starts with legal drug and proceeds to illegal drugs [29]. Above all, Shashemene is a town in which Rastafarians view Ethiopia as a promised land live. Cannabis use is a common practice among Rastafarians which brought a major challenge to both youth and law enforcement in the town [30].

Even though there is a huge burden associated with cannabis use disorder among cannabis users in general and young adults in particular, there is a shortage of study in sub-Saharan Africa and also in our country. Therefore, this study assessed the magnitude of cannabis use disorder and associated factors among cannabis young adult users at Shashemene Town.

## 2. Methods and Materials

### 2.1. Study Setting, Study Design, Participants, and Sampling Procedure

A cross-sectional study was conducted from May to June 2016 at Shashemene Town which is located at a distance of 250 km south of Addis Ababa. The town is a center for trade where Rastafarians, who consider Ethiopia a holly and promised land, had developed a settlement on a land offered by Emperor Haile Selassie. All young adults using cannabis aged 18–25 and permanent residents were included in the study. The sample size was determined using single proportion formula and found to be 423, and participants were selected using exponential discriminative snow ball sampling.

### 2.2. Data Collection Tools, Quality Control Issues, and Study Variables

Data was collected by five trained members of youth club using WHO standard ASSIST and SRQ questionnaires prepared in English, translated into Amharic, and then back-translated into English to keep its consistency. Alcohol, Smoking, and Substance Involvement Screening Tool (ASSIST) is a WHO standard tool that identifies use of substances and associated problems. It was found to be reliable in different countries of the world including Zimbabwe. SRQ is also a screening tool that measures CMD and it is validated in Ethiopia. The overall questionnaires were composed of background information and specific information which cover open and close ended questions about cannabis use and associated factors to collect relevant information needed. Pretest was conducted on 5% of the total sample size in Arsi Negele town young adults who use cannabis, and necessary correction was taken after the pretest done on the questionnaires.

The questionnaires are designed to be conducted by interview. Thus, each data collector identified one participant and oriented respondents about the ethical principles of confidentiality and data management prior to involvement with data collection. Once the participant fulfilled the inclusion criteria she/he heard a letter of information and signed the informed consent, to participate on research. Then the data was collected through interview and used to recruit two or more participants; then only one participant was taken randomly. The pattern was continued until sufficient sample has been identified.

The data collectors checked the completeness of the information during data collection period. Throughout the course of the data collection period regular meetings were held between the data collectors and the principal investigator in order to solve any problem encountered during data collection process. The collected data was reviewed and checked for completeness before data entry; the incomplete data was discarded. Data entry format template was produced and programmed.

The study variables included were individual level variables such as sociodemographic characteristics; psychosocial factors such as family history of substance use, peer pressure, easily availability of cannabis, price of cannabis, and childhood sexual abuse; clinical variable which is common mental disorder; and behavioral variables such as cigarette smoking, drinking alcohol, chewing khat, shisha use, and cannabis use disorder.

### 2.3. Data Management and Analysis

Coded variables were entered into EPI Info 2000 version 5 which was transported into SPSS version 20 Window software program for cleaning and analysis. Descriptive statistics (frequencies, median, and percentages) were used to describe sociodemographic and other independent variables of the study population and cannabis dependence. The variables that are found to be significant at the 5% level were entered into the multivariable logistic regression model using the enter method and at a 95% confidence interval to determine the actual predictors for cannabis dependence.

### 2.4. Ethical Considerations

Ethical clearance was obtained from the Ethical Review Committee of Training and Research Department. The proposal has passed through the ethical clearance process. A formal letter was obtained from institutional reviewed board of Gondar University and submitted to the respective subcity administration and permission letter was obtained to conduct the study before data collection period.

After explaining confidentiality of information, informed consent has been obtained from the study participants. Privacy of the study participants was maintained at the time of administering the questionnaires. The right of respondents was kept by giving them freedom to interrupt the interview whenever they want. Participants were priorly informed that after termination of the project, all questionnaires will be destroyed. Therefore, there will be no related information of any participant. Study participants who were found to have either cannabis use disorder or CMD or both were referred to Shashemene General Hospital.

## 3. Result

### 3.1. Sociodemographic Characteristics of the Respondents

From the total of 423 study participants recruited, 415 were included in the study making the response rate of 98.1%. The age of the respondents ranges between 18 and 25 with the mean age (±SD) of 22.51 (±2.27) years. The respondents were predominantly 309 (74.5%) of male sex. Concerning educational status of the respondents, more than half, 252 (60.7%), of them were 9–12th grade while 131 (31%) of them were 1–8th grade. In occupational status 124 (29.9%) of the respondents were daily laborers followed by private employees which were 119 (28.7%). Almost three-fourth, 309 (74.5), of them were single. Most, 262 (63%), of the respondents were Orthodox religion followers, and about one-third, 145 (34.9%), of them were Oromo in ethnicity. More than half, 249 (60%), of them were living with family (Table 1).

### 3.2. Substance Related Factors

From the entire sample majority 400 (96.4%) of the respondents reported having used khat, about 377 (90%) reported having used alcohol, and 365 (88%) reported having used cigarette within the last three months. Shisha use within the last three months was reported by 28% of the total respondents (Table 2).

### 3.3. Psychosocial and Clinical Factors

Majority, 324 (78%), of the respondents had easy access to cannabis and 296 (71.3%) of the respondents believed that cannabis is cheap. Most, 289 (69.6%), of the respondents attributed their cannabis use to peer pressure. Parental substance use was reported by 289 (40%) of the study participants. Childhood sexual abuse was also reported by 24 (5.3%) of the respondents. According to SRQ scoring system of CMD among the entire respondents about two-third, 275 (66.26%), of them experienced common mental disorder in the past four weeks (Table 3).

### 3.4. Prevalence of Cannabis Use Disorder among Respondents at Shashemene Town, Oromia Region, Ethiopia, 2016

**Figure 1:**
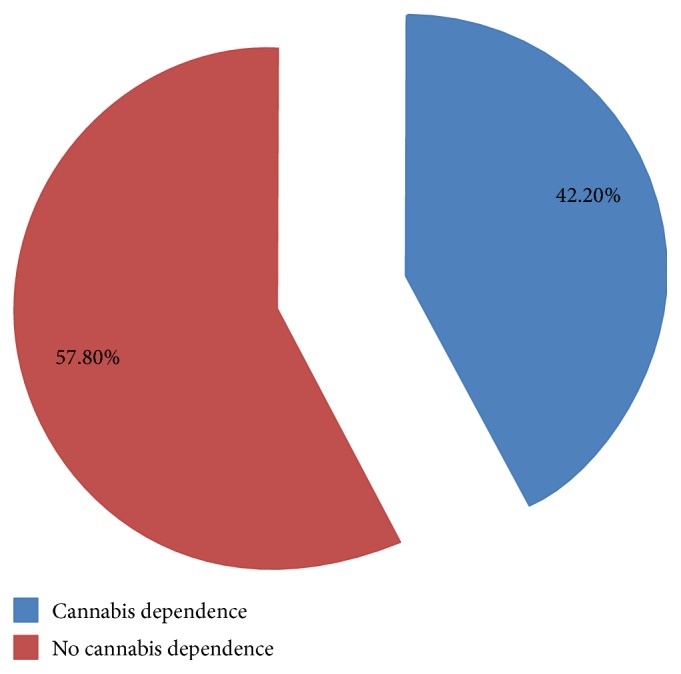
Prevalence of cannabis use disorder among young adults at Shashemene Town, Oromia Region, Ethiopia, 2016.

According to ASSIST scoring system of cannabis disorder, the overall prevalence of cannabis use disorder was 42.2% (Figure 1).

### 3.5. Factors Associated with Cannabis Use Disorder

To determine the association of independent variables with cannabis dependence, bivariate logistic regression analysis was done. The crude analysis was done by including sociodemographic factors, psychosocial factors, clinical factors, behavioral factors, and outcome variable, that is, cannabis use disorder.

Upon bivariate logistic regression analysis, sociodemographic characteristics including sex, job, marital status, and living condition; psychosocial factors including parental substance use, peer pressure, easy access to cannabis, and sexual abuse; clinical factor CMD; and behavioral factors including tobacco use, alcohol use, khat use, and shisha use had significant association.

Variables with *p* value < 0.2 in the bivariate logistic regression analysis were taken to multivariate logistic regression analysis to see whether there is significant association or not by controlling confounding factors. During the multivariate logistic regression current cigarette smoking, current shisha use, and CMD were found to have significant association with cannabis use disorder.

Accordingly the odds of cannabis use disorder for the respondents who reported having smoked cigarette in the last three months were 6.1 times (AOR = 6.12, 95% CI: 2.13–17.57) more compared to respondents who did not smoke cigarette in the last three months. The odds of cannabis use disorder in respondents who used shisha in the last three months were 5.6 times (AOR = 5.59, 95% CI: 3.50–8.93) higher than the respondents who did not use shisha in the last three months. The odds of cannabis use disorder for the respondents who reported to have CMD were 2.12 times (AOR = 2.12, 95% CI: 1.22–3.71) more compared to respondents who did not have CMD (Table 4).

## 4. Discussion

Cannabis use disorder is a problematic pattern of cannabis use leading to clinically significant impairment or distress. According to ASSIST scoring system of cannabis use disorder, the overall prevalence of cannabis use disorder was 42.2%. This study is in line with the findings from USA and two different studies in Netherland and their prevalence was 38.5%, 42.2%, and 40%, respectively [11, 12, 14].

However, it was higher when compared with many other studies in USA, Canada, West Europe, Australia, New Zealand, India, Namibia, Zimbabwe, and Rwanda in which the prevalence rate was in the range of 1.3–15.3%. In the study done in USA, the prevalence was 1.6, which is lower than the current study; this could be because the study done in USA was on general population through household survey; then from this general population survey, they extracted the data specific to young adults. On the top of this, the possible reason could be the difference in study period which was from 2012 to 2013 [13]. In the study done in Canada, the prevalence was 5%, which is lower than this study; the possible reason could be that the study populations were students and the setting was school in addition to the socioeconomic variation [10]. The study conducted in Rwanda shows 2.54% prevalence, which is lower than this study, and the possible reasons could be the age range of the study participants from 14 to 35 and the measurement tool which is Cannabis Abuse Screening tool [18]. The prevalence was also lower (3.26%) in India and possible reasons could be study participants and setting (patients in the hospital), the tool being diagnostic tool, and the study period being from 2011 to 2012 [17]. The other possible reasons in overall variation in the prevalence might be accounted for the socioeconomic and cultural difference across the studies [10, 13, 17, 18].

On multivariate logistic regression analysis, using tobacco and shisha in the past three months and experiencing CMD in the last four weeks were found to have significant association with cannabis use disorder.

The odds of cannabis use disorder among respondents who use tobacco in the last three months were six times (AOR: 6.1, 95% CI: 2.13–17.575) higher compared to respondents who were not smoking tobacco in the past three months. The possible explanation might be that tobacco smokers are more likely to normalize using other psychoactive substances, particularly cannabis, which makes them use cannabis heavily and as a result they become prone to develop cannabis use disorder.

The finding of this study was in line with other studies conducted in United Kingdom which showed that tobacco was the driver of cannabis use disorder in young adults [27]. The finding also further supported by the gateway drug theory that works based on the concept that the sequence of first time use is not random but shows trends or the pattern of substance use that starts with legal drugs and proceeds to illegal drugs. Particularly, tobacco precedes the use of cannabis [29].

The odds of cannabis use disorder among respondents who used shisha in the past three months were about 4.3 times (AOR: 4.313, 95% CI: 2.427–7.664) higher compared to respondents who were not used shisha in past three months.

The same explanation given to tobacco can be applicable to explain how using shisha can lead to cannabis use disorder. Even though shisha and tobacco have similarity in terms of ingredients and their effect on the consumers, they are quite different in terms of setting where they are used; as a result shisha users may have different factors which can make them vulnerable to cannabis use and dependence [28, 29].

The odds of cannabis use disorder among respondents with CMD were 2.12 times (AOR: 2.125, 95% CI: 1.218–3.708) higher compared to respondents who were not having CMD. This makes the present study in agreement with the previous studies in Australia that confirmed that the odds of cannabis use disorder among depressed participants are higher than nondepressed participants [22, 25, 26]. Possible explanation for the high degree of cannabis use disorder found in young adult who get depressed or anxious might be self-medicating the preexisting depressive or anxiety symptoms by using cannabis. As is known most people tend to use psychoactive substances including cannabis heavily when they get depressed or anxious in order to get relief from their unpleasant feeling as a result of depression or anxiety. This is more pronounced when the one who became depressed or anxious is young adult; this is also supported by self-medication hypothesis that explains how CMD leads a person to initiate and maintain psychoactive substance use and abuse.

In conclusion, the prevalence of cannabis use disorder among young adults at Shashemene was higher than that reported by many studies done worldwide. Having common mental disorders has an increased likelihood of cannabis use disorder. In addition to common mental disorders, cannabis use disorder was highly associated with cigarette use as well as shisha use.

## Figures and Tables

**Table 1:** Sociodemographic characteristics of respondents at Shashemene Town, Oromia Region, Ethiopia, 2016.

Characteristics	Frequency	Percentage
*Age (mean)*	22.51 (±SD 2.27)	
*Sex *		
Male	309	74.5
Female	106	25.5
*Educational status *		
Elementary	131	31.6
High school	252	60.7
Others	32	7.7
*Occupational status *		
Private employee	119	28.7
Daily laborer	124	29.9
Jobless	56	13.5
Student	99	23.9
Other	17	4.1
*Marital status *		
Single	309	74.5
Married	64	15.4
Divorced	22	5.3
Other	20	4.8
*Religion*		
Muslim	103	24.8
Orthodox	262	63.2
Protestant	35	8.4
Other	15	3.6
*Ethnicity*		
Oromo	145	24
Amhara	92	22.2
Gurage	82	19.8
Tigre	44	10.6
Wolayta	35	8.4
Other	17	4
*Living condition*		
Alone	166	40
With family	249	60

**Table 2:** Magnitude of other substances use in the last three months among the respondents at Shashemene Town, Oromia Region, Ethiopia.

Characteristics	Frequency	Percentage
Khat		
Yes	400	96.4
No	15	3.6
Alcohol		
Yes	377	90
No	38	10
Cigarette		
Yes	365	88
No	50	12
Shisha		
Yes	118	28.4
No	297	71.6

**Table 3:** Psychosocial and clinical factors of respondents at Shashemene Town, Oromia Region, Ethiopia, 2016.

Characteristics	Frequency	Percentage
Easily access cannabis		
Yes	324	78.1
No	91	21.9
Believe cannabis is cheap		
Yes	296	71.3
No	119	28.7
Peer pressure		
Yes	289	69.6
No	126	30.4
Parental substance use		
Yes	166	40
No	249	60
Childhood sexual abuse		
Yes	24	5.8
No	391	94.2
CMD		
Yes	275	66.27
No	140	33.73

**Table 4:** Multivariate logistic regression analysis results of factors associated with cannabis dependence among young adults at Shashemene Town, Oromia Region, Ethiopia 2016.

Explanatory variable	Cannabis use disorder		COR 95% CI	AOR 95% CI
	No	Yes		
Sex				
Male	196	113		
Female	44	62	2.44 (1.56–3.83)	1.11 (0.61–2.01)
Living condition				
Alone	160	89		
With family	80	86	1.93 (1.3–2.88)	1.12 (0.68–1.86)
Marital status				
Single	192	117		
Married	33	31	1.54 (0.9–2.36)	1.03 (0.53–2.01)
Other	15	27	2.95 (1.51–5.78)	1.57 (0.69–3.56)
Job				
Private employee	64	55		
Daily laborer	62	62	1.16 (0.70–1.93)	1.12 (0.62–2.01)
Jobless	27	29	1.250 (0.66–2.36)	1.72 (0.81–3.64)
Student	80	19	0.28 (0.15–.51)	.531 (0.263–1.07)
Other	7	10	1.66 (0.59–4.66)	2.00 (0.60–6.64)
Easy access to cannabis				
No	59	32		
Yes	181	143	1.46 (0.89–2.24)	0.89 (0.48–1.65)
Peer pressure				
No	82	44		
Yes	158	131	1.54 (1.00–2.38)	1.48 (0.85–2.56)
Parental substance use				
No	155	94		
Yes	85	81	1.57 (1.57–2.34)	0.88 (0.53–1.45)
Sexual abuse				
No	234	157		
Yes	6	18	1.57 (1.74–11.51)	1.86 (0.64–5.42)
CMD				
No	104	36		
Yes	136	139	2.95 (1.89–4.61)	2.12 (1.22–3.71)^*∗∗∗*^
Cigarette smoking				
No	45	5		
Yes	195	170	7.846 (3.04–20.21)	6.118 (2.13–17.57)^*∗∗∗*^
Drinking alcohol				
No	32	6		
Yes	208	169	4.33 (1.77–10.61)	1.85 (0.67–5.12)
Chewing khat				
No	12	3		
Yes	228	172	3.02 (0.84–8.94)	0.76 (0.17–3.36)
Using shisha				
No	206	91		
Yes	34	84	5.59 (3.50–8.94)	4.31 (2.43–7.66)^*∗∗∗*^

^*∗∗∗*^ *p* = 0.0001.
